# Regulatory roles of osteopontin in human lung cancer cell epithelial‐to‐mesenchymal transitions and responses

**DOI:** 10.1002/ctm2.486

**Published:** 2021-07-08

**Authors:** Lin Shi, Jiayun Hou, Lin Wang, Huirong Fu, Yiwen Zhang, Yuanlin Song, Xiangdong Wang

**Affiliations:** ^1^ Department of Pulmonary and Critical Care Medicine Zhongshan Hospital Shanghai China; ^2^ Institute for Clinical Science Shanghai China; ^3^ Shanghai Institute of Clinical Bioinformatics Shanghai China; ^4^ Shanghai Engineering Research for AI Technology for Cardiopulmonary Diseases Shanghai China; ^5^ Jinshan Hospital Centre for Tumor Diagnosis and Therapy Shanghai China; ^6^ Fudan University Shanghai Medical College Shanghai China

**Keywords:** EMT, lung cancer, metastasis, OPN, PI3K

## Abstract

**Background:**

Lung cancer is still the main cause of death in patients with cancer, due to poor understanding of intracellular regulations. Of those, osteopontin (OPN) may induce the epithelial‐to‐mesenchymal transition (EMT) to promote tumor cell metastasis. The present study aims to evaluate the regulatory mechanism of internal and external OPN in the development of lung cancer.

**Methods:**

We evaluated genetic variations and different bioinformatics of genes in chromosome 4 among subtypes of lung cancer using global databases. We validated the expression of OPN and EMT‐related proteins (e.g., E‐cadherin, vimentin) in 208 non‐small‐cell lung cancer (NSCLC) tumors and the adjacent nontumorous tissues, further to explore the function of OPN in the progression of lung cancer, with a focus on a potential communication between OPN and EMT in the lung cancer.

**Results:**

We found that OPN might act as a target molecule in lung cancer, which is associated with lymph node metastasis, postresection recurrence/metastasis, and prognosis of patients with lung cancer. Biological behaviors and pathological responses of OPN varied among diseases, challenges, and severities. Overexpression of OPN was correlated with the existence of EMT in lung cancer tissues. Internal and external OPN plays the decisive roles in lung cancer cell movement, proliferation, and EMT formation, through the upregulation of OPN‐PI3K and OPN‐MEK pathways. PI3K and MEK inhibitors downregulated the process of EMT and biological behaviors of lung cancer cells, probably through altering vimentin‐associated cytoskeletons.

**Conclusion:**

OPN can be a metastasis‐associated or specific biomarker for lung cancer and a potential target for antimetastatic treatment.

## Graphical abstract

1. OPN can be a metastasis‐associated or specific biomarker for lung cancer.
2. OPN over‐expressed in lung cancer was correlated with cell movement, proliferation and the existence of EMT.
3. OPN plays the decisive roles in lung cancer biological behaviors and EMT formation, through PI3K/Akt and MAPK/Erk1/2 pathways.

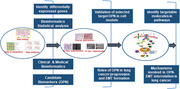

## 1 INTRODUCTION

Lung cancer has gradually become the main cause of cancer‐related death worldwide in recent years,^1^ mainly including lung adenocarcinoma (ADC), squamous cell carcinoma (SCC), large cell carcinoma (LCC), and small‐cell lung carcinoma (SCLC). Despite improvements in surveillance and clinical treatment strategies, the 5‐year survival rate of lung cancer is still low,^2^ which is largely due to the aggressive malignant tumor with high risk of distant metastasis after therapy. Profiles of gene expression were mapped to explore the diagnostic biomarkers and therapeutic targets of lung cancer through detection of specific genes belonging to subtypes of lung cancer.^3^, ^4^ The interaction of complex networks and multiple targeting oncogenic pathways may be more important than targeting a single oncogene^5^ related to lung cancer metastasis.

The formation of EMT plays a critical role in cancer metastasis as the early change in cell morphology and transcriptional and/or phenotypic changes of epithelial and mesenchymal cell during tumorigenesis.^6^ During EMT process, epithelial cells lose the polarity and acquire new features of mesenchyme involved in wound healing, fibrosis, fetal development, embryogenesis, and tissue remodeling.^7^, ^8^, ^9^ Normal polarized epithelial cells with the expression of distinct proteins, for example, E‐cadherin, claudins, occludins, and cytokeratins, may exhibit a “cobblestone” homophilic morphology. Cancer cells may develop a fibroblastoid morphology and mesenchymal phenotypes characterized by specific biomakers, such as vimentin, actin, fibronectin, and N‐cadherin.^10^ EMT occurred in tumor microenvironment induced by hypoxia and cigarette smoke^11^, ^12^ through activation of many signaling pathways (TGF‐β or OPN‐related signaling pathway).^13^, ^14^ OPN contributes to processes of tissue remodeling and EMT during tumor progression, for example, renal tubular epithelial cells, hepatocellular carcinomas, or breast carcinomas.^15^, ^16^ Our previous studies demonstrated that OPN gene and protein overexpressed in acute exacerbation of chronic obstructive pulmonary disease and lung cancer are probably responsible for evolution and heterogeneity of lung cancer.^17^, ^18^ OPN overexpression was considered as an independent prognostic factor in patient overall survival and associated with more aggressive phenotypes of patients with lung cancer,^19^ although the molecular mechanisms are still unclear.

The current study aims to identify global genetic variations and different bioinformatics (e.g., molecular function and biological processes) of genes in chromosome 4 among subtypes of lung cancer as potential candidates of biomarkers and evaluate prediction value OPN in ADC and SCC for the prognosis of patients. We investigated the existence of OPN and EMT in fresh NSCLC tumor tissues using tissue microarrays and explored the biological functions of OPN in lung cancer in vitro and in vivo to understand the regulatory role of OPN in EMT process in lung cancer. Furthermore, we investigated the involvement of phosphoinositide 3‐kinase (PI3K)/Akt and mitogen‐activated protein kinase (MAPK)/Erk1/2 signaling pathways and PI3K subunits in EMT induced by OPN, the potential roles of OPN‐PI3K‐EMT on lung cancer cell movement and proliferation, and potential values as therapeutic approach to lung cancer.

## 2 METHODS

### 2.1 Selection of target molecules

The keywords of “human,” “metastasis,” and “lung cancer” were used in different GEO and GSE databases. Twenty‐seven of 504 GSE datasets contained comparative profiles of gene expression among different subtypes of lung cancer and the adjacent nontumorous lung tissues. We found 21 GSE datasets containing lung cancer (including ADC, SCC, LCC, and SCLC) and metastasis, and the data normalizing methods in 16 GSE datasets were employed in our study, including 140 SCC, 537 ADC, 56 SCLC, nine LCC, and 590 noncancerous cases (Table 1).

**TABLE 1:** Comparative gene express datasets in GEO database (cases)

GEO series	ADC	SCC	LCC	SCLC	Noncancer
GSE10072	56	0	0	0	49
GSE12472	0	35	0	0	28
GSE18842	14	32	0	0	45
GSE19804	60	0	0	0	60
GSE1987	7	17	0	0	7
GSE29249	3	3	0	0	6
GSE31552	35	25	0	0	66
GSE32665	87	0	0	0	92
GSE3268	0	5	0	0	5
GSE32863	58	0	0	0	58
GSE33356	60	0	0	0	60
GSE40275	8	4	2	25	43
GSE43458	80	0	0	0	30
GSE4824	26	4	6	22	8
GSE6044	16	15	0	9	5
GSE7670	27	0	1	0	28
Total	537	140	9	56	590

Abbreviations: ADC, lung adenocarcinoma; LCC, large‐cell carcinoma; SCC, squamous cell carcinoma; SCLC, small‐cell lung carcinoma.

### 2.2 Data standardization and reorganization

Data from each dataset of gene expression profiling were normalized against five reference genes: actin beta (ACTB), non‐POU domain containing octamer‐binding (NONO), lactate dehydrogenase A (LDHA), phosphoglycerate kinase 1 (PGK1), and aldolase A fructose‐bisphosphate (ALDOA). All data of gene expression from datasets were normalized with their reference genes. All data were later integrated with 1559 genes containing official symbols in chromosome 4 of lung cancer. Gene expressions in different subtypes of lung cancer were compared with noncancerous cases to identify genes that are significantly up‐ or downregulated for two‐folds or greater than two‐folds. Lung cancer subtype‐specific genes were defined by comparing the difference among subtypes. Gene ontology (GO) gene function annotation and gene cluster analysis were evaluated using Molecule Annotation System (MAS 3.0) and GenCLip 2.0.^20^

### 2.3 Survival analyses

Survival prediction values and the univariate associations between expression profiles and survivals of selected target gene OPN in patients with ADC or SCC were analyzed by Cox regression. Then, we used the survdiff function of the “survival” package to assess the differences between log rank *p*‐values and survival curves. The normalized RNA‐seq data were pulled from 532 SCC samples and 528 ADC samples through the Broad GDAC FIREHOSE on November 7, 2013. Clinical survival data were obtained from The Cancer Genome Atlas (TCGA) FTP server on December 6, 2013. The survival models were also built by the data of 1715 NSCLC samples from 10 independent datasets.^21^

### 2.4 Patients and specimens

Specimens containing lung tumors and the adjacent nontumorous lung tissues were obtained with the informed consent of 208 patients who underwent a curative resection and mediastinal lymph node dissection for NSCLC from Zhongshan Hospital Affiliated to Fudan University, Shanghai, China in 2005. All specimens were processed with paraffin blocks based on the availability of suitable formalin‐fixed, paraffin‐embedded tissue as well as the complete clinicopathologic date with follow‐up on all patients. Pathological classification was assessed according to World Health Organization criteria, and tumor/node/metastasis (TNM) staging was determined based on the 8th edition of International Union Against Cancer. The overall survival includes two parts, one is the interval between surgery and the last observation of the surviving patients, and the other is the interval between surgery and death. The follow‐up lasted until July 2010, and the median follow‐up is about 43 months (range 1–66). Ethical approval (B2019‐232R) was obtained from the Zhongshan Hospital Research Ethics Committee.

### 2.5 Cell lines and reagents

Human normal bronchial epithelial cells (HBE), human monocytic cells (U937), and human lung cancer cells (A549) were obtained from Shanghai Chinese Academy of Science. Cells were cultured into the RPMI 1640 complete medium (HyClone, Logan, USA) supplemented with 10% fetal bovine serum (FBS; Biowest, Nuaillé, France) and 1% penicillin‐streptomycin antibiotics (PS; Genom, Hangzhou, China) in 5% CO_2_ at 37°C. Hypoxic culture was performed in the three‐phase incubator at 37°C, 1% O_2_, 5% CO_2_, and 90% relative humidity. Human recombinant OPN (R&D, Shanghai, China), PI3K/Akt specific inhibitor SHBM1009 (Biovision, San Francisco, USA), Erk1/2 specific inhibitor PD98059 (Biovision, San Francisco, USA), mouse monoclonal anti‐vimentin antibody (Abcam, Hong Kong, China), and rabbit polyclonal to OPN (Abcam, Hong Kong, China) were used in this study. Rabbit monoclonal anti‐p‐Akt, p‐Erk1/2, total‐Akt, total‐Erk1/2, and E‐cadherin were purchased from Cell Signaling Technology Company (Danvers, MA). *Cell‐IQ* live cell imaging platform was manufactured by Chipmantech (Tampere, Finland).

### 2.6 Cigarette smoke extract

Cigarette smoke extract (CSE) was produced in accordance with the previous methods.^22^ The 60‐ml syringe with 3‐ml trimmed straw was connected together to make a smoking device with a rubber band. The smoke from cigarette (Hongyun Honghe Tobacco, Kunming, China) without the filter was drawn by this smoking device and injected into a 50‐ml tube with 5 ml of 10% FBS and 1% PS. The initial smoke extract was filtered into a 15‐ml centrifuge tube with a 0.22‐μm filter in an ultraclean table, of which 100 μl were placed in one well of the 96‐well plate and repeated six times, followed by the addition of 100 μl RPMI 1640 with 10% FBS and 1% PS. The concentration of CSE was measured by the absorbance value at the wavelength of 320 nm. The average value of the two liquids was reduced and diluted to 1.158 as 100% CSE.

### 2.7 Animals

Male athymic BALB/c nu/nu mice aged 4–6 weeks were purchased from the Central Laboratory of Animal Science at Fudan University (Shanghai, China) and housed with water and food ad libitum, under specific pathogen‐free conditions in laminar‐flow cabinets. All animal experiments were approved by Shanghai Medical Experimental Animal Care Committee and conducted according to the care and use of laboratory animals guidelines of National Institutes of Health (NIH; Bethesda, MD, USA).

### 2.8 Tissue microarray analysis

Tissue microarrays were performed as previously described.^23^ Briefly, OPN antibody (1:200), vimentin antibody (1:100) and E‐cadherin antibody (1:100) were used to detect the protein expression of OPN, vimentin, and E‐cadherin. Additionally, the expression of OPN was semi‐quantitatively determined by two pathologists independently. Staining intensity was divided into four grades, 0, 1, 2, or 3, while the intensity ranged from negative and weak to strong intensity. The percentage was defined for the four grades, where the lowest grade 0 was given 0%, the middle grades 1 and 2 were scored as 1%–33% and 34%–66%, respectively, and the highest grade 3 as 67%–100%. The sum of the intensity scores was used as the final staining scores, which are defined from 0 to 2 as low expression and from 3 to 6 as high expression.

### 2.9 Measurements of gene expression

The gene expression was measured by quantitative RT‐PCR with an ABI 7000 PCR instrument (Eppendorf, Hamburg, Germany). Briefly, cells were treated by a guanidinium isothiocyanate/chloroform‐based technique (Trizol, Life Technologies). RNA was isolated and determined at OD 260 nm and subsequently reverse transcribed to cDNA according to the directions of the kit (Invitrogen, USA). Primer (Invitrogen) concentrations (10 nM) in a total volume of 20 μl before use were optimized with SYBR Green PCR master kit. Finally, the gene expression level was measured by two‐stage program parameters: 1 min at 95°C, followed by 40 cycles of 5 s at 95°C and 30 s at 60°C. The data were standardized according to GAPDH expression and all data were expressed as calibrated raw threshold cycle values after normalization. Primer sequences are shown in Table S1.

### 2.10 Measurement of target proteins

To measure the signal pathway of EMT induced by OPN, A549 cells (1 × 10^5^ cells/well) were counted and cultured in six well plates for 24 h and pretreated with PD98059 at 0.1, 1, and 10 μM and SHBM1009 at 0.01, 0.1, and 1 μM for 2 h, and then treated with OPN (500 ng/ml) or vehicle for 20 min. The proteins were extracted, followed by denaturation and quantification, and separated by 10% SDS‐PAGE gels (Beyotime, Shanghai, China). Then, proteins were transferred to polyvinylidene fluoride membranes, followed by blocking with 5% defatted milk for 2 h, rinsing, and incubating with primary antibodies at 4°C overnight. Primary antibodies were removed and labeled with the corresponding secondary antibodies for 2 h. After treatment, bands were visualized by Electro‐Chemi‐Luminescence and exposed by X‐ray film. OPN‐associated effects on the phosphorylation of Akt and Erk1/2 were evaluated and the expression of OPN, E‐cadherin, and vimentin proteins in NSCLC tissues and A549 cells was assayed with Phoretix 1D software.

### 2.11 Immunofluorescence staining

A549 cells (1 × 10^4^ cells/ml) were seeded onto sterile coverslips placed in 24‐well cell culture plates for 24 h. Immunofluorescence staining was performed in A549 cells treated without or with OPN at a concentration of 5, 50, and 500 ng/ml for 48 h. A549 cells were fixed (4% paraformaldehyde) and permeabilized (0.1% Triton X‐100) for 20 min. Then, the cells were blocked in PBS containing 10% goat serum for 30 min after washing and incubated with vimentin antibody (1:200) and E‐cadherin antibody (1:200) overnight. Each chamber was washed three times (5 min each time), and incubated with the corresponding secondary antibodies for 1 h at 37°C. After washing with 4′,6′‐diamidino‐2‐phenylindole for 5 min, the fluorescence density was examined by immunofluorescence microscope (Olympus/BX51, Tokyo, Japan) in dark conditions.

### 2.12 Cell migration and movement

Cells were trypsinized and resuspended, and then plated in the 8‐μm upper chambers (Corning, MA, USA) at a concentration of 10^5^ cells in 200‐μl serum‐free medium of 0.1% BSA, whereas mediums with or without OPN and 10% FBS were added to the lower chambers. The number of migrating cells was counted 24 h after the incubation and removal of the nonmigrating cells from the upper surface. Cells on the membranes for the migration assays were fixed with 4% paraformaldehyde, stained with Giemsa (Sigma), and counted under inverted microscope and photographed under 200× magnification. Cell movement was evaluated using the scratch assay in each well with 5 × 10^5^ cells after nonadherent cells were removed. Then, cells were incubated with serum‐free medium for 0, 24, and 48 h. Experiments were performed in triplicates and three random fields of inverted microscope images were counted for each experiment.

### 2.13 Cell proliferation

Cell proliferations were analyzed using Cell Counting Kit‐8 Cell (CCK8; Dojindo, Kumamoto, Japan). A549 cells at 2 × 10^3^ cells per well were cultured in 96‐well plates with or without PI3K inhibitor SHBM1009 and Erk1/2 inhibitor PD98059 for 2 h in advance, and then cultured with 500 ng/ml OPN for 24, 48, and 72 h. According to the CCK8 protocol, cells in each well were incubated with CCK‐8 reagent (10 μl) for 2 h in 5% CO_2_ at 37°C and estimated the fluorescence at 450 nm by a microplate reader (Bio‐Rad Laboratories Inc., CA, USA). The cell movement was detected after treatment with small interfering RNA (siRNA; GenePharma, Shanghai, China), OPN (Sigma), OPN antibody (Santa Cruz), or CSE. The cell proliferation index was calculated as a percentage of absorbance of the treated group in relation to that of the untreated control cells.

### 2.14 siRNA and transfection

Three different sense and antisense strands of OPN‐specific siRNA (Table S1) were provided by Ribobio (Guangdong, China). Cells were transfected with vehicle, nonspecific siRNA or OPN‐specific siRNA according to Lipofectamine 2000 instructions (Invitrogen, USA). The transfection efficiency was verified by real‐time PCR and Western blot. The most effective cells were selected to construct stable interference cell lines for transwell assays and in vivo studies.

### 2.15 Alive measurement of cell bio‐behaviors

Cell bio‐behaviors, including the total number of cells, cell morphology, and cell movement, were measured by the real‐time cell monitoring system *Cell‐IQ* platform (Chip‐Man Technologies, Tampere, Finland). Images were taken at about 5‐min intervals for 72 h. Analysis was performed with vision technology to monitor and record time‐lapsed data. Morpho‐types and cell movement were analyzed and quantified. For each group, six to 12 replicate images were randomly selected and analyzed.

### 2.16 In vivo tumor growth assay

Nude mice were allocated into three groups at random (*n* = 5/time point/group) and in vivo tumor growth was evaluated by the subcutaneous injection of 1 × 10^7^ cells with vehicle, nonspecific siRNA or OPN‐specific siRNA into nude mice. We monitored tumor growth by observing and recording the volume of the tumor; *V* (mm^3^) = width^2^ (mm^2^) × length (mm)/2. After 6 weeks of inoculation, the mice were sacrificed and tumors were harvested and weighed. The expressions of OPN, EMT‐related proteins, PI3K/Akt, and MAPK/Erk1/2 in vivo were detected by immunohistochemical assay and Western blot.

### 2.17 Statistical analysis

Values among different lung cancer subtypes were statistically compared by Student's *t*‐test and Mann–Whitney *U*‐test. The associations between clinical phenomes and OPN expression were examined with *χ* ^2^ tests. The cumulative survival time was calculated by the Kaplan–Meier method and analyzed by the log‐rank test. Data were represented as a mean ± standard error of the mean (SEM). Differences between groups were assessed by the Student's *t*‐test after ANOVA analyses. Statistical significance level was set as .05.

## 3 RESULTS

### 3.1 Global screening of target genes in lung cancer subtypes

**FIGURE 1:**
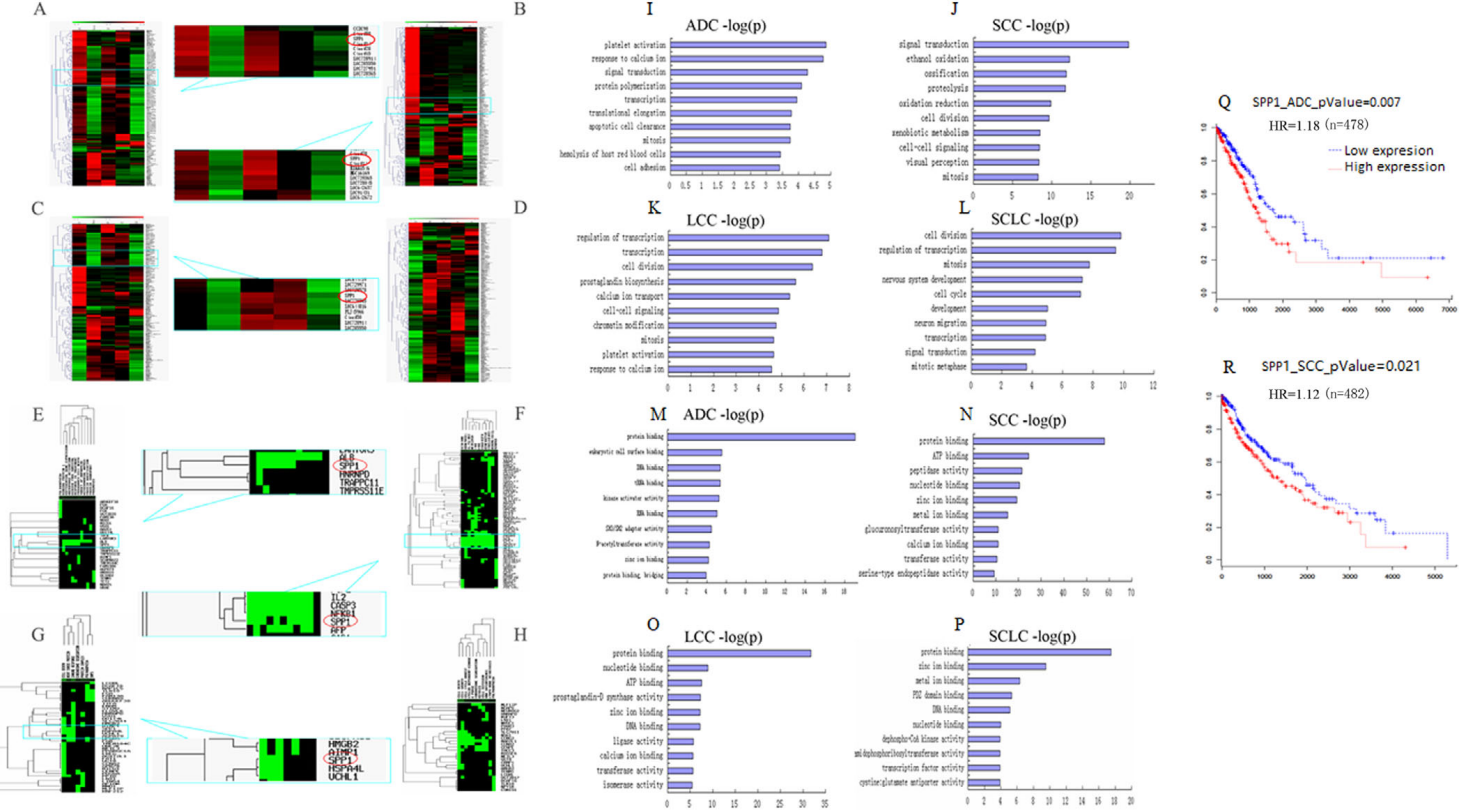
Global screening of target genes in lung cancer subtypes. Gene expressions with more than two‐fold up‐ or down‐changes (A–D), alterations of target gene clusters (E–H), signaling pathways (I–L), and biological functions (M–P) in patients with lung adenocarcinoma (ADC), squamous cell carcinoma (SCC), large cell carcinoma (LCC), and small‐cell lung carcinoma (SCLC). Poor prognosis was associated with high expression of OPN gene (SPP1) in patients with ADC (Q) or SCC (R). Values among different lung cancer subtypes were statistically compared by Student's *t*‐test and Mann–Whitney *U*‐test. The associations between clinical phenomes and OPN expression were examined with *χ* ^2^ tests. The cumulative survival time was calculated by the Kaplan–Meier method and analyzed by the log‐rank test

To explore genetic variations of genes in chromosome 4 among different subtypes of lung cancer, we measured the number of genes with more than a two‐fold change (up/down) with significance, as compared with adjacent normal tissues, in patients with ADC (Figure 1A), SCC (Figure 1B), LCC (Figure 1C), or SCLC (Figure 1D). The numbers are 134/53, 236/110, 120/90, and 46/302, respectively. Overexpressed genes were associated with binding protein 1 in the extracellular matrix of ADC and SCC, cell death and chromosome segregation in LCC and SCLC, and M phase cell cycle arrest in SCC and SCLC. Overexpressed genes were also associated with macrophage activation, disease‐free survival, response to treatment, protein transport, and prognosis in ADC (Figure 1E); DNA replication, calcium channels, S phase, estrogen receptor, and histone deacetylase in SCC (Figure 1F); immune response, heat shock protein, and SNPs in LCC (Figure 1G); or cell growth, mitosis, cell division, RNA interference, and lymph node metastasis in SCLC (Figure 1H). We noticed that the expression of OPN gene (SPP1) was significantly upregulated in cell function clusters in three subtypes of NSCLC.

### 3.2 Gene function clustering and prognosis prediction

In GO biological process, genes related to the mitosis and signal transduction overexpressed in all four subtypes of lung cancer (Figure 1I–L); genes for transcription in ADC, LCC, and SCLC; platelet activation and response to calcium ion in ADC and LCC; and cell division in SCC, LCC, and SCLC. Overexpressed genes in biological processes also included protein polymerization, apoptotic cell clearance, and cell adhesion in ADC (Figure 1I); proteolysis, ethanol oxidation, oxidation reduction, and ossification in SCC (Figure 1J); chromatin modification and prostaglandin biosynthesis in LCC (Figure 1K); and nervous system development, cell cycle, neuron migration, and mitotic metaphase in SCLC (Figure 1L).

The molecular functions of overexpressed genes mainly included protein binding and zinc ion binding in all four subtypes of lung cancer (Figure 1M–P); nucleotide binding in SCC (Figure 1N), LCC (Figure 1O), and SCLC (Figure 1P); DNA binding in ADC (Figure 1M), LCC, and SCLC; metal ion binding in SCC and SCLC; ATP binding, transferase activity, and calcium ion binding in SCC and LCC; genes with tRNA binding, kinase activator activity, RNA binding, and SH3/SH2 adaptor activity in ADC; peptidase activity, glucuronosyl transferase activity in SCC; ligase activity and isomerase activity in LCC; and PD2 domain binding, transcription factor activity, and dephospho‐COA kinase activity in SCLC. Of those, OPN was mainly involved in transcription, cell adhesion, ossification, and protein binding. In addition, overexpression of OPN predicted poor overall survival in NSCLC with 1.18 hazard ratio (HR) in ADC (*p *= .007, Figure 1Q) or 1.12 in SCC (*p *= .02, Figure 1R).

### 3.3 Validation of selected target OPN in cell models

**FIGURE 2:**
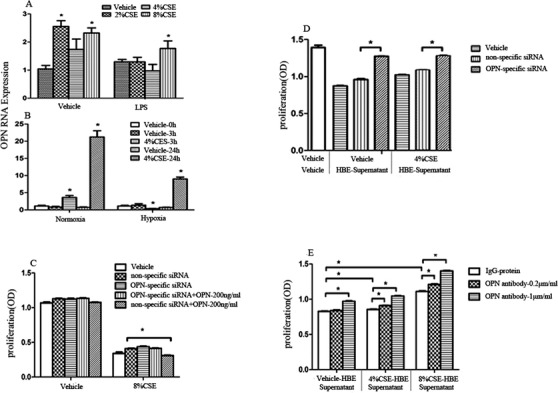
Validation of selected target OPN in cell models. OPN gene expression of human bronchial epithelial cells with vehicle and LPS (A) or with normoxia and hypoxia (B) after challenge with CSE at different concentrations and different times. The proliferation rates of cells treated with nonspecific siRNA or OPN‐specific siRNA with or without external OPN challenged with 8% CSE (C). The proliferation rates of U937 cells treated with supernatant from cell^OPN−^ and cell^OPN+^ stimulated with CSE (D), or with co‐culture of external OPN with supernatants harvested from 4% and 8%‐stimulated cells for 24 h (E). Data are represented as mean ± SEM. Differences between groups were assessed by the Student's *t*‐test, after ANOVA analyses

**FIGURE 3:**
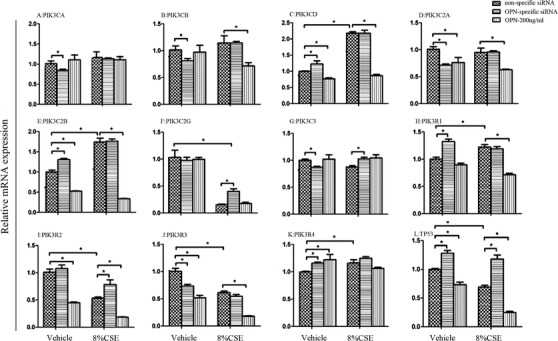
Influence of internal and external OPN in PIK3 subtypes and TP53. mRNA expression of PIK3CA (A), CB (B), CD (C), C2A (D), C2B (E), C2G (F), C3 (G), R1 (H), R2 (I), R3 (J), R4 (K), and TP53 (L) in cells treated with nonspecific siRNA or OPN‐specific siRNA and external OPN after challenge with vehicle or 8% CSE. Data are represented as mean ± SEM. Differences between groups were assessed by the Student's *t*‐test, after ANOVA analyses

We validated OPN gene expression of HBE under various challenges and decisive roles of OPN in cell proliferation. OPN gene expression was upregulated after CSE at different concentrations, which was downregulated by adding LPS (Figure 2A) or hypoxia (Figure 2B). High concentration (8%) of CSE significantly inhibited epithelial proliferation of cells infected with nonspecific siRNA lentivirus (cell^OPN+^) or OPN‐specific siRNA lentivirus (cell^OPN−^) treated with or without external OPN, among which we observed that cell^OPN+^ was more sensitive to external OPN (Figure 2C). In order to explore roles of secreted OPN from CSE‐stimulated cells, we harvested the supernatant from cell^OPN+^ or cell^OPN−^ stimulated with 4% CSE for 24 h and applied the supernatant to the culture of fresh lymphocytes (U937). The supernatant from CSE‐stimulated cell^OPN+^ could inhibit U937 cell proliferation significantly, as compared with that from cell stimulated with vehicle or cell^OPN−^ (Figure 2D). The addition of external OPN antibody prevented the reduction of cell proliferation caused by the supernatant from CSE‐stimulated cell^OPN+^ in a dose‐dependent pattern (Figure 2E). We further evaluated the influence of internal and external OPN in variation of intracellular signal signatures like PI3K family members and P53 in cell^OPN+^, cell^OPN−^, or cell^OPN+^ with OPN protein at 200 ng/ml (Figure 3). The expression of PIK3CA (Figure 3A), CB (Figure 3B), and C3 (Figure 3G) reduced and CD (Figure 3C), C2B (Figure 3E), R1 (Figure 3H), and TP53 (Figure 3L) increased in cell^OPN−^, as compared with those in cell^OPN+^. The expression of PIK3CD, C2B, R2, and TP53 reduced in cell^OPN+^ treated with OPN protein. The expression of PIK3C2A (Figure 3D) and R3 (Figure 3J) reduced, while R4 (Figure 3K) increased in both cell^OPN−^ and cell^OPN+^ with OPN protein. After stimulation with 8% CSE, PIK3CD, C2B, R1, and R4 were overexpressed, and C2G (Figure 3F), R2 (Figure 3I), and R3 downexpressed in both cell^OPN+^ and cell^OPN−^, while the expression of C2G, R2, and TP53 in cell^OPN−^ was significantly higher than that in cell^OPN+^. External OPN protein could downregulate the expression of PIK3CB, CD, C2A, C2B, C2G, R1, R2, R3, and TP53 in cell^OPN+^ after CSE stimulation. Our data demonstrated that OPN gene expression increased after stimulation with CSE, and both external and internal OPN protein could regulate the genes of PI3K isoforms in epithelial cells after challenge.

### 3.4 OPN–EMT association in NSCLC cells and tissues

**FIGURE 4:**
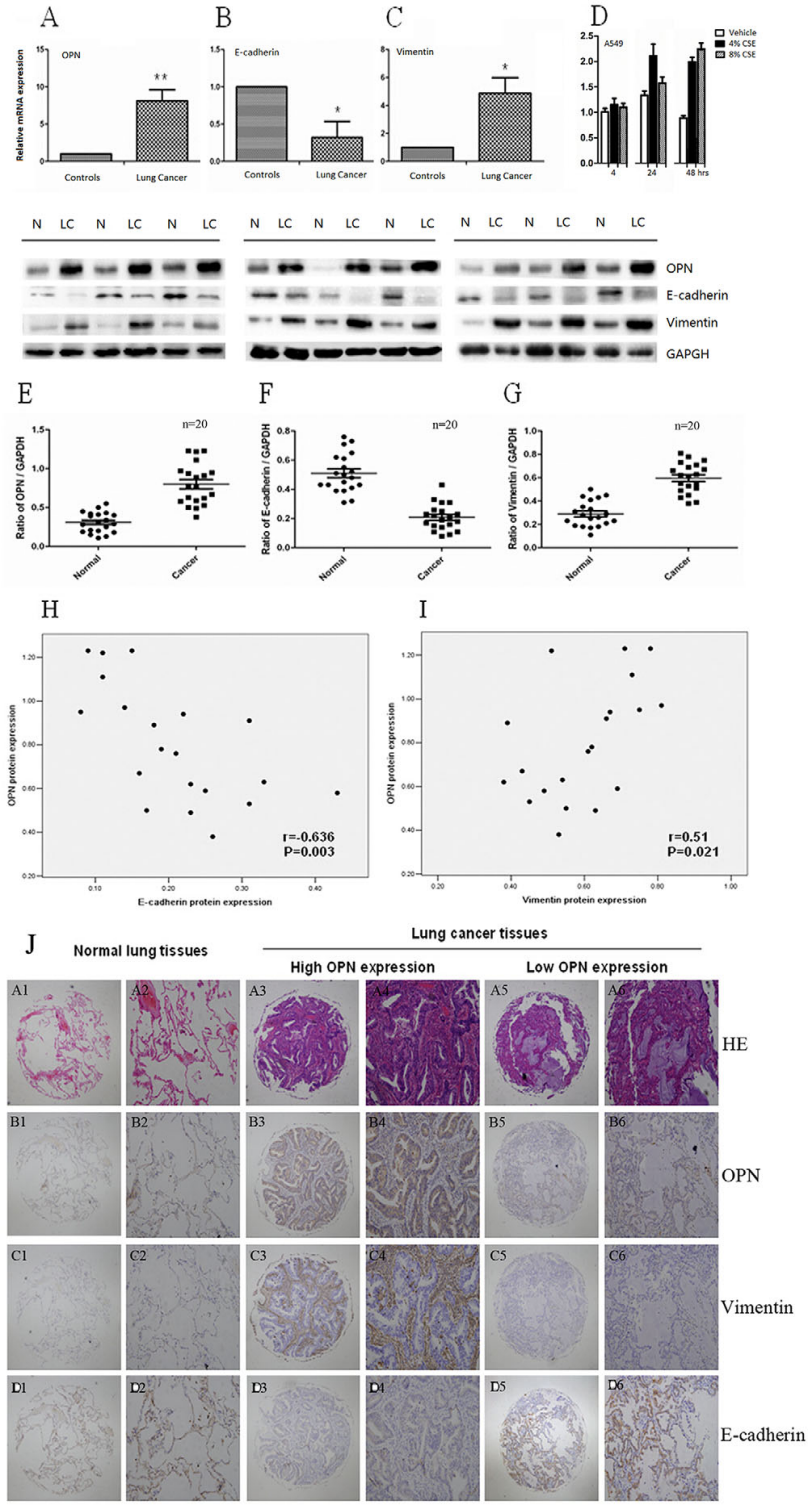
Validation of OPN–EMT association in NSCLC cells and tissues. mRNA of OPN (A), E‐cadherin (B), and vimentin (C) expressed in lung cancer tissue (LC) and adjacent normal tissue as controls (N), and OPN mRNA in A549 cells 4, 24, and 48 h after challenges with vehicle, or 4% and 8% CSE (D). The scatter plot of OPN (E), E‐cadherin (F), and vimentin (G) in NSCLC tissues and adjacent normal tissues. The OPN expression was negatively and positively correlated with E‐cadherin (H) and vimentin protein levels (I), respectively. Immunohistochemistry staining of NSCLC tissue microarrays from 208 patients at different clinical stages and histologic types of NSCLC were divided into high‐ and low‐OPN expression groups (J), including hematoxylin‐eosin staining (HE, A1–6), OPN (B1–6), vimentin (C1–6), and E‐cadherin (D1–6) (A1–D1, A3–D3, A5–D5 ×40; A2–D2, A4–D4, A6–D6 ×100). Data are represented as mean ± SEM. Differences between groups were assessed by the Student's *t*‐test, after ANOVA analyses. * and ** stand for *p‐*values less than .05 and .01, respectively, in comparison with controls

We detected the mRNA and protein expression of OPN and EMT‐related proteins (including E‐cadherin and vimentin) in 20 fresh NSCLC tumors and their adjacent normal lung tissues. The expression of OPN (Figure 4A) and vimentin (Figure 4C) in NSCLC tissues was significantly higher than that in adjacent normal lung tissues, while the expression of E‐cadherin decreased in NSCLC tissues (Figure 4B). OPN gene expression was upregulated from 24 h after treatment with 4% CSE and 8% CSE (Figure 4D). The scatter plot of OPN, E‐cadherin, and vimentin protein expression revealed a significant negative correlation between OPN and E‐cadherin in NSCLC tissues (*r* = −0.636, *p *= .003; Figure 4E,F,H) and a positive correlation between OPN and vimentin protein levels (*r* = 0.51, *p *= .021; Figure 4E,G,I). Immunohistochemistry staining demonstrated the positive staining for OPN in the cytoplasm, and high levels of OPN and vimentin staining, and low level of E‐cadherin in NSCLC tissues (Figure 4J).

### 3.5 Association of OPN with postoperative recurrence or metastasis

High expression of OPN accounted for 48% (99 of 208 patients) of specimens from NSCLC patients, with significant correlations between OPN expression and clinical phenomes (Table 2). The expression level of OPN was closely related to lymph node metastasis in patients with NSCLC (*p *= .01) before operation, and with the postoperative recurrence or metastasis of NSCLC to extrapulmonary organs (*p *= .03). Total 5‐year survival rate of patients with high and low OPN expression was 52% (109/208) (*p = *.351).

**TABLE 2:** Association between OPN expression and clinicopathologic variables in 208 patients with non‐small‐cell lung cancer

		OPN expression		
Variables	No. of patients	Low	High	*p*
Age				
≤60	111	62	49	.286
>60	97	47	50	
Gender				
Male	147	79	68	.549
Female	61	30	31	
Smoking status				
Smokers	84	46	38	.575
Nonsmokers	124	63	61	
Histological type				
SCC	85	38	47	.173^a^
ADC	110	60	50	
Others^b^	13	11	2	
Differentiation				
Well/moderate	115	62	53	.628
Poor	93	47	46	
Tumor size				
≤3 cm	66	34	32	.861
>3 cm	142	75	67	
Lymph node metastasis				
Yes	90	38	52	.010
No	118	71	47	
Pathological stage				
I–II	144	79	65	.287
III–IV	64	30	34	
Recurrence or metastasis				
Yes	92	40	52	.033^c^
No	84	50	34	
Unknown	32	19	13	

^a^ *p*‐Values reflect comparisons between SCC and ADC.

^b^ Others include adenosquamous carcinoma, large cell carcinoma, mucoepidermoid carcinoma, and carcinosarcoma.

^c^ *p*‐Values reflect comparison between patients with and without recurrence or metastasis.

### 3.6 External OPN effects in lung cancer cell EMT

**FIGURE 5:**
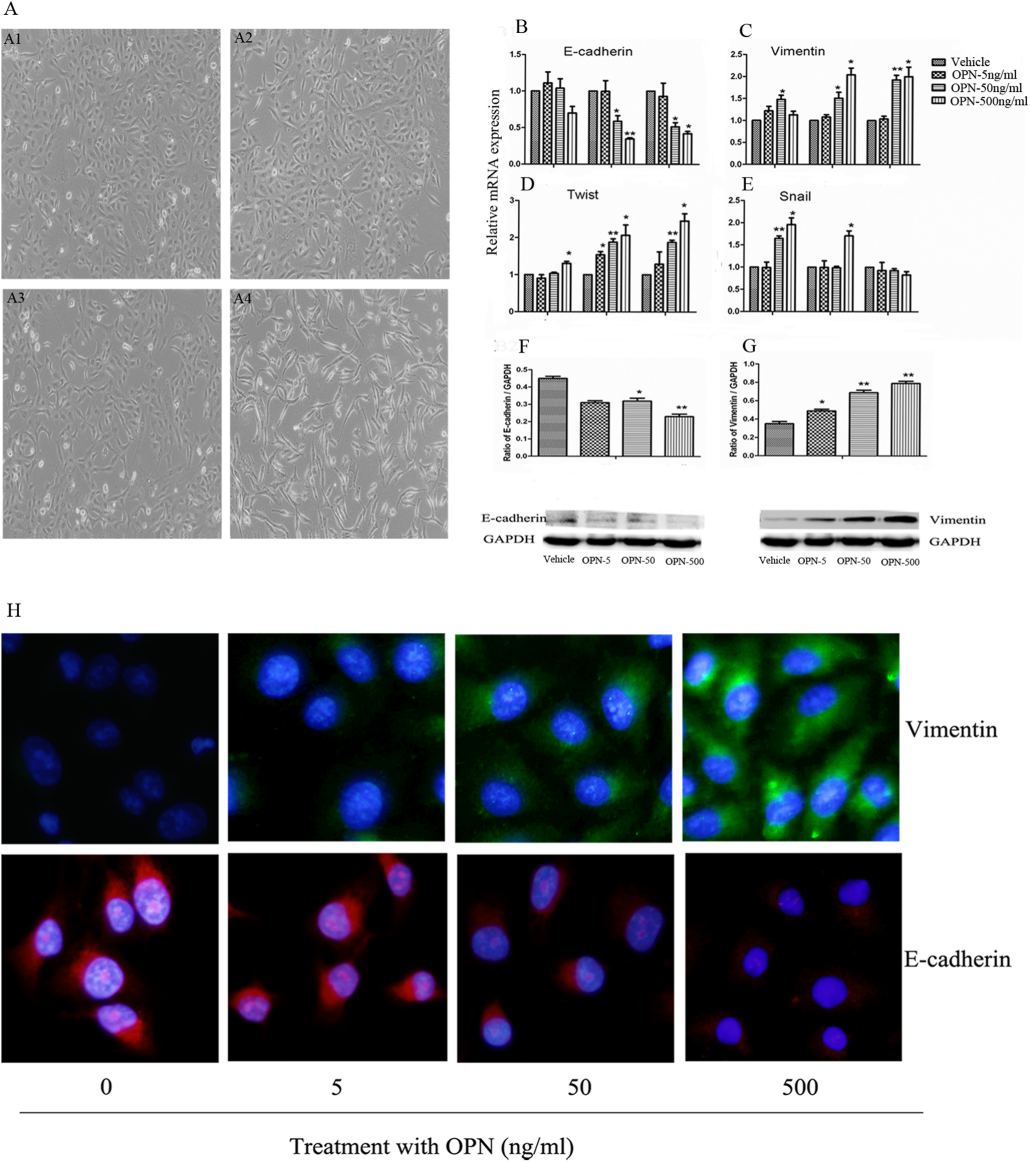
Validation of external OPN effects in lung cancer cell EMT. The epithelial morphology and growth pattern of A549 cells were observed 48 h after being treated with vehicle (A1) or OPN at 5, 50, or 500 ng/ml (A2, A3, or A4), respectively. The mRNA expression of E‐cadherin (B), vimentin (C), Twist (D), and Snail (E) was measured 24, 48, and 72 h after challenge with vehicle or external OPN protein at 5, 50, and 500 ng/ml, respectively. The expression of E‐cadherin (F) and vimentin (G) proteins was measured 48 h after OPN treatment at different doses, respectively, and stained by immune‐fluorescence staining (H). Data are represented as mean ± SEM. Differences between groups were assessed by the Student's *t*‐test, after ANOVA analyses. * and ** stand for *p‐*values less than .05 and .01, respectively, in comparison with vehicle

Dynamic effects of external OPN protein in the development of lung cancer cell EMT were investigated in A549 cells within 72 h after treatment with OPN protein at different concentrations. The “cobblestone” morphology of A549 cells (Figure 5A1) became a fibroblast‐like morphology where many cells lost contact with their neighboring cells at 48 h after treatment with OPN at 5, 50, or 500 ng/ml (Figure 5A2–4), which was more obvious when the concentration was 500 ng/ml. To characterize the induction of OPN in EMT phenotypes, E‐cadherin, vimentin, and corresponding transcription factors Snail and Twist were monitored dynamically after treatment with external OPN protein. The mRNA expression of E‐cadherin decreased (Figure 5B) and vimentin increased (Figure 5C), respectively, from 48 h after treatment with OPN protein at 50 and 500 ng/ml. Additionally, the mRNA expression of Twist increased from 24 h after OPN at 500 ng/ml and from 48 h after OPN at 5 and 50 ng/ml (Figure 5D), and Snail increased 24 h after OPN at 50 ng/ml and 24 and 48 h after OPN at 500 ng/ml (Figure 5E), as compared with vehicle (*p *< .05 or less, respectively). The expression of E‐cadherin and vimentin proteins significantly decreased and increased, respectively, at 48 h after OPN stimulation in a dose‐dependent pattern (Figure 5F,G), which was also demonstrated by immunofluorescence staining (Figure 5H).

### 3.7 Roles of OPN in lung cancer cell biological function

**FIGURE 6:**
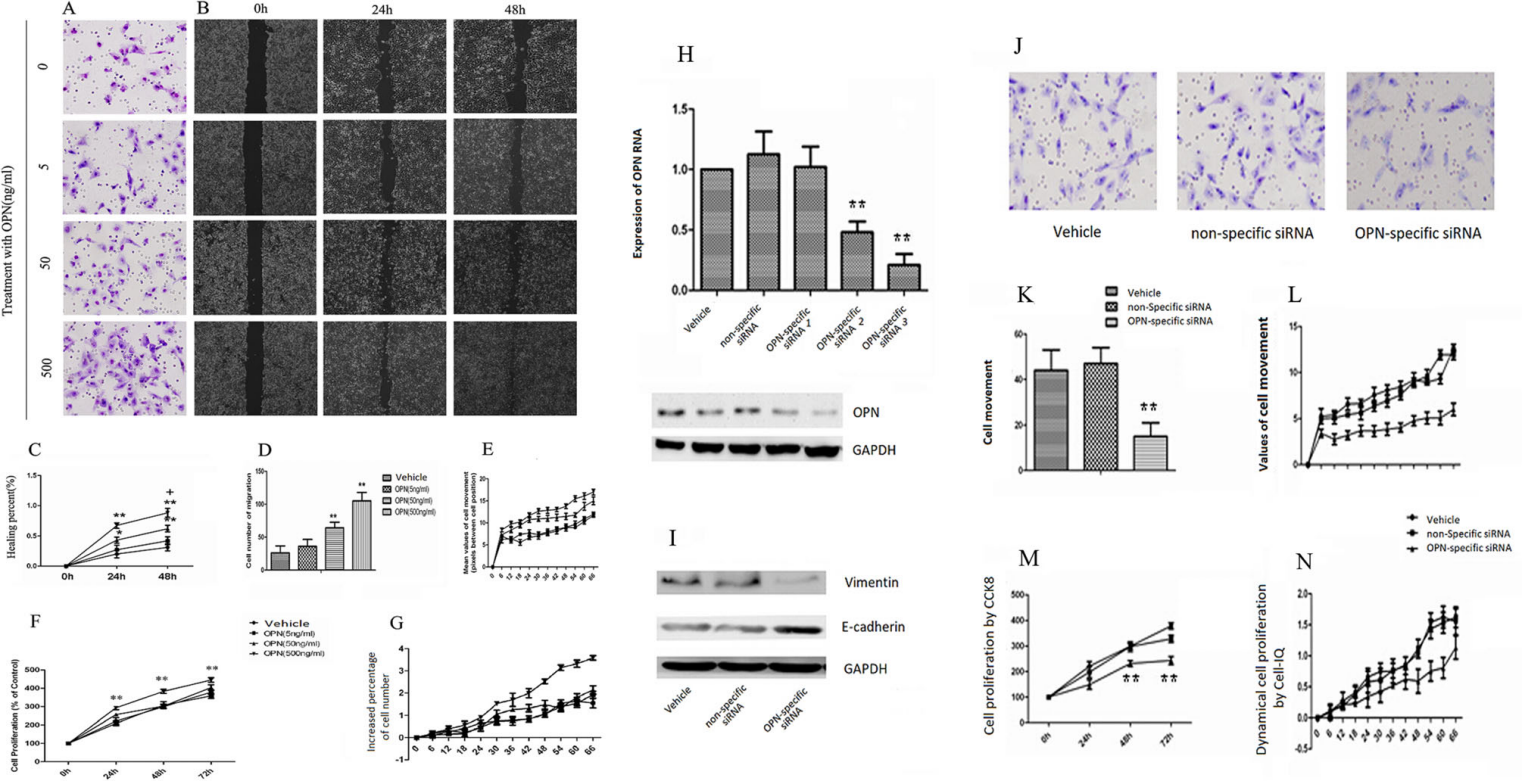
Roles of OPN in lung cancer cell migration and proliferation. Cell number (A), migration (B), healing percentage (C), and number of migrated cells from upper to lower chambers in transwell (D) increased after OPN at 50 and 500 ng/ml. Dynamical cell proliferation was detected by *Cell‐IQ* monitoring (E) and CCK8 test (F), and dynamical cell movement by *Cell‐IQ* monitoring (G). Role of internal OPN in lung cancer cell proliferation was investigated by OPN‐specific siRNA, which inhibited the expression of OPN mRNA and production of OPN (H). Protein expression of high E‐cadherin and low vimentin were found in cell^OPN−^ (I). We noticed cell^OPN−^ had significantly lower capacity of cell movement from upper to lower chambers in transwell (J, K) and dynamical movement detected by *Cell‐IQ* monitoring (L), as well as cell proliferation by CCK8 (M) and by *Cell‐IQ* monitoring (N). Data are represented as mean ± SEM. Differences between groups were assessed by the Student's *t*‐test, after ANOVA analyses.* and ** stand for *p‐*values less than .05 and .01, in comparison with vehicle, and + and ++ stand for *p‐*values less than .05 and .01, as compared with OPN‐treated cells at 24 h, respectively

We validated the roles of internal and external OPN in biological function of lung cancer cells and found that OPN increased cell number (Figure 6A), migration (Figure 6B), and healing percentage (Figure 6C) of A549 cells in a dose‐ and time‐dependent patterns. The number of migrated cells from upper to lower chambers in transwell increased after treatment with OPN at 50 and 500 ng/ml (Figure 6D). Dynamical cell movement increased from 24 to 30 h after treatment with OPN at 500 ng/ml (Figure 6E). Dynamical proliferation of A549 cells significantly increased from 6 h after OPN treatment, detected by CCK8 test (Figure 6F) and *Cell‐IQ* monitoring (Figure 6G). Roles of internal OPN in lung cancer cell proliferation was investigated by OPN‐specific siRNA, which inhibited the expression of OPN mRNA and production of OPN (Figure 6H). We noticed that cell^OPN−^ had significantly lower capacity of cell movement from upper to lower chambers in transwell (Figure 6J,K) and dynamical movement detected by *Cell‐IQ* monitoring (Figure 6L), as well as cell proliferation detected by CCK8 (Figure 6M) and by *Cell‐IQ* monitoring (Figure 6N). High E‐cadherin and low vimentin protein expressions were found in cell^OPN−^ (Figure 6I).

### 3.8 OPN‐induced EMT through PI3K and MEK pathways

**FIGURE 7:**
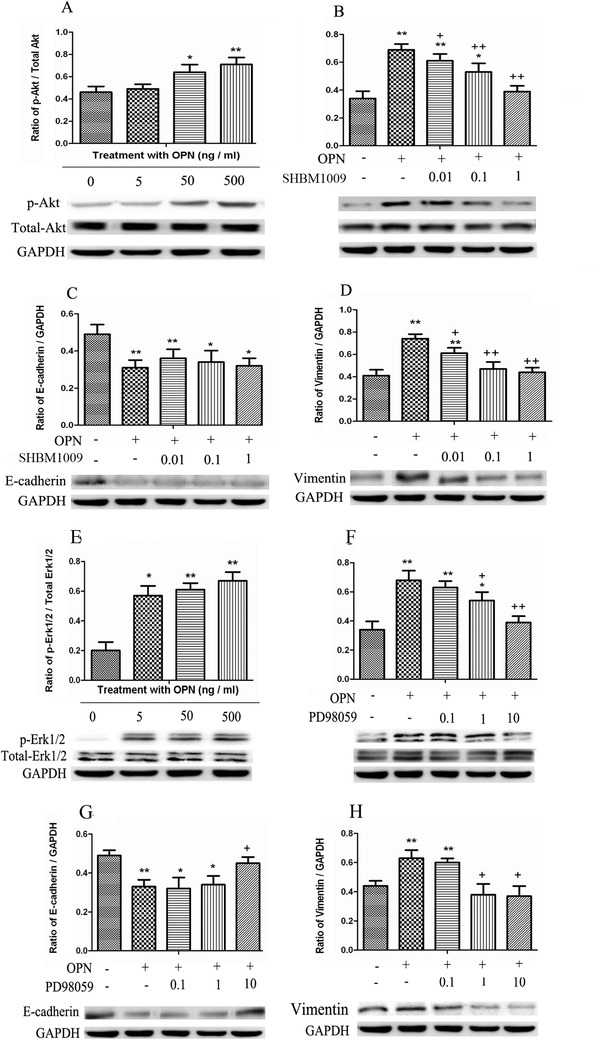
Roles of PI3K/Akt and MAPK/Erk1/2 on OPN‐induced EMT. Total Akt and p‐Akt were detected after challenge with OPN at different concentrations (5, 50, or 500 ng/ml) (A), as well as for cells treated with 0.01, 0.1, or 1 μM of the PI3K inhibitor SHBM1009 (B). E‐cadherin (C) and vimentin (D) were measured 48 h after challenge with 500 ng/ml OPN and 0.01, 0.1, or 1 μM of the PI3K inhibitor SHBM1009. Total Erk1/2 and p‐Erk1/2 were detected after challenge with OPN at different concentrations (5, 50, or 500 ng/ml) (E), as well as for cells treated with 0.1, 1, or 10 μM of the Erk1/2 inhibitor PD98059 (F). E‐cadherin (G) and vimentin (H) were measured 48 h after challenge with 500 ng/ml OPN and 0.1, 1, or 10 μM of the PD98059. Data are represented as mean ± SEM. Differences between groups were assessed by the Student's *t*‐test, after ANOVA analyses.* and ** stand for *p‐*values less than .05 and .01 as compared with vehicle, and + and ++ stand for *p*‐values less than .05 and .01, as compared with OPN challenge, respectively

On basis of evidence that OPN influenced biological function of normal HBE (Figures 2 and 3), we investigated potential mechanisms by which OPN influences lung cancer cells and found that p‐Akt increased from 20 min after treatment with 50 and 500 ng/ml OPN (Figure 7A). A549 cells pretreated with PI3K/Akt inhibitor SHBM1009 for 2 h had significantly lower responses to OPN stimulation (Figure 7B). SHBM1009‐pretreated cells also had low expression of vimentin protein after OPN stimulation, especially when cells were pretreated with SHBM1009 at high dose (Figure 7D), whereas there was no difference of E‐cadherin expression between cells pretreated with vehicle and with SHBM1009 (Figure 7C). After treatment with OPN at 5 ng/ml, p‐Erk1/2 significantly increased (Figure 7E), while PD98059‐pretreated cells had lower levels of p‐Erk1/2 in a dose‐dependent pattern (Figure 7F). Pretreatment with PD98059 at 10 μM could prevent OPN‐induced down‐ or up‐expression of E‐cadherin (Figure 7G) or vimentin (Figure 7H).

### 3.9 OPN induced lung cancer cell migration and metastasis

**FIGURE 8:**
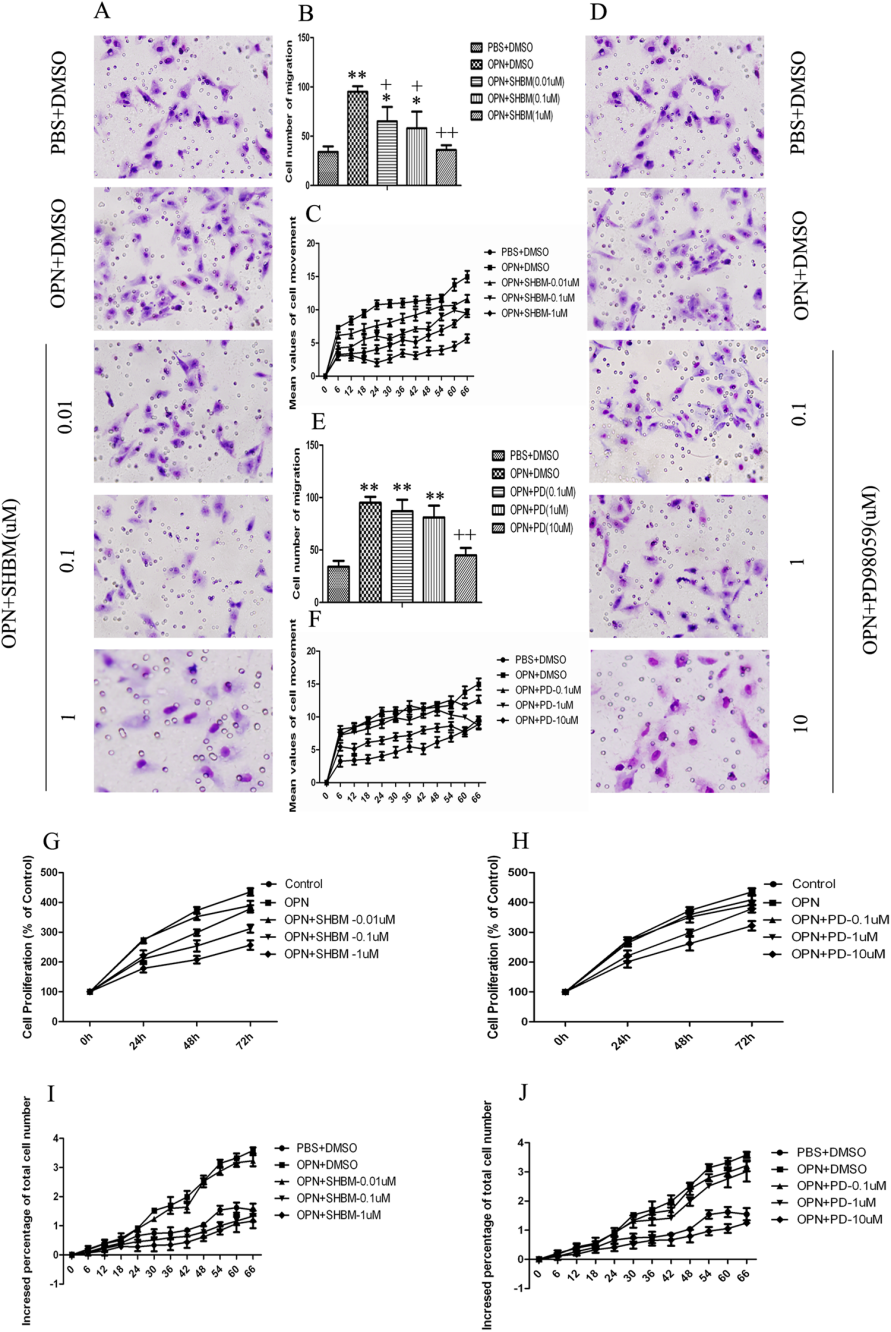
OPN promotes cell migration and proliferation. Cell migration (A and B) was detected by transwell chambers after challenge with OPN with different concentrations of PI3K inhibitor SHBM1009 (0.01, 0.1, or 1 μM), and the dynamical movements (C) were detected by *Cell‐IQ* monitoring. Cell migration (D and E) was detected by transwell chambers after challenge with OPN with different concentrations of Erk1/2 inhibitor PD98059 (0.1, 1, or 10 μM), and the dynamical movements (F) were detected by *Cell‐IQ* monitoring. Dynamical cell proliferation was measured by CCK8 (G) and *Cell‐IQ* monitoring (I) in cells pretreated with OPN with different concentrations of SHBM1009. Dynamical cell proliferation was measured by CCK8 (H) and *Cell‐IQ* monitoring (J) in cells pretreated with OPN with different concentrations of PD98059. Data are represented as mean ± SEM. Differences between groups were assessed by the Student's *t*‐test, after ANOVA analyses. * and ** stand for *p‐*values less than .05 and .01, in comparison with untreated control cells, and + and ++ stand for *p‐*values less than .05 and .01, as compared with OPN‐treated cells at 24 h, respectively

**FIGURE 9:**
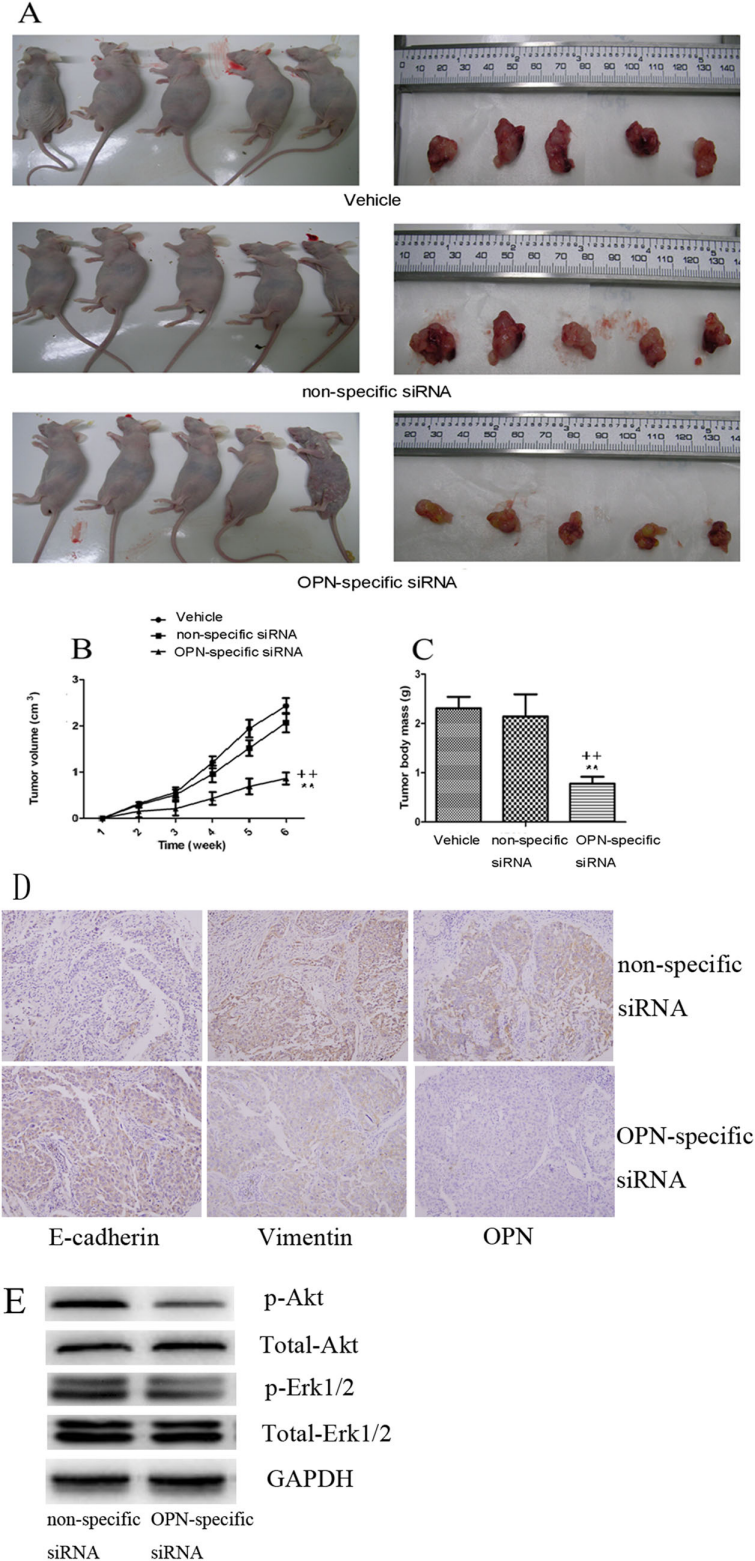
OPN‐specific siRNA inhibits lung cancer xenograft growth in vivo. In vivo tumor growth in OPN‐specific siRNA mice was significantly inhibited compared with vehicle or nonspecific siRNA, whereas no significant difference in tumor volumes between vehicle or nonspecific siRNA (A and C) was found. Tumor growth curves were plotted during the experiment (B). Expression of E‐cadherin, vimentin, and OPN in nonspecific siRNA or OPN‐specific siRNA mice was detected by immunohistochemistry staining (D). Expressions of PI3K/Akt and Erk1/2 signaling pathway components were detected in the tumor from mice determined by Western blot. Data are represented as mean ± SEM. Differences between groups were assessed by the Student's *t*‐test, after ANOVA analyses.** stands for *p‐*values less than .01 as compared with vehicle, and ++ stands for *p*‐values less than .01, as compared with nonspecific siRNA

We noticed that SHBM1009 inhibited A549 cell migration induced by external OPN (Figure 8A,B) and dynamical movements detected by *Cell‐IQ* monitoring (Figure 8C) in a dose‐dependent pattern. The OPN‐increased cell number migration (Figure 8D,E) significantly reduced in cells pretreated with PD98059 at 10 μM. Dynamical movement of cells pretreated with PD98059 at 1 and 10 μM decreased from 6 h after culture with OPN (Figure 8F). Cell proliferation (Figure 8G,H) measured by CCK8 and dynamical cell proliferation (Figure 8I,J) by *Cell‐IQ* monitoring in cells pretreated with SHBM1009 at 0.01 μM and PD98059 at 1 and 10 μM decreased from 24 h after OPN stimulation. A549 cells without any infection, infected with nonspecific siRNA lentivirus (cell^OPN+^), or infected with OPN‐specific siRNA lentivirus (cell^OPN−^) were subcutaneously implanted into nude mice, respectively. In addition to the observation of tumor growth (Figure 9A), we also found that tumor volumes significantly increased by time after implant of lung cancer cells pretreated with vehicle or cell^OPN+^, while animals implanted with cell^OPN−^ had significantly lower tumor volumes from 4 weeks after the implant as compared with animals implanted with cell^OPN+^ (Figure 9B, *p *< .01). Tumor masses in animals with cell^OPN−^ at 6 weeks were significantly smaller than those in animals with cell^OPN+^ (Figure 9C, *p *< .01). The level of E‐cadherin was high and vimentin was low in animals implanted with cell^OPN−^ (Figure 9D). The expressions of p‐Akt and p‐Erk1/2 was downregulated in tumor tissues from the animals implanted with cell^OPN−^ (Figure 9E).

## 4 DISCUSSION

NSCLC accounts for nearly 85% of lung cancers and is an aggressive malignance with high mortality rate.^24^, ^25^ NSCLC is prone to distant metastasis through loss of cell adhesion, increase in ability of migration, and invasion into new distant tissues and colonization. The understanding of migration and metastasis of lung cancer cells is critical in the early detection, prevention, and improvement of metastasis and outcome of patients.^26^ OPN‐dominated signal pathways were considered as one of the critical roles in the development and heterogeneity of lung cancer and were responsible for lung cancer cell sensitivity to target therapies, rapid progression, and complex metastasis.^27^ OPN‐dominated molecular networks were also found to play an important role in lung tissue injury and repair and might be the decisive factor of therapeutic interaction between telocytes and stem cells.^28^ During inflammation, OPN could increase leukocyte migration into inflammatory loci through multifactor regulations, for example, monocyte chemoattractant protein 1, β1 integrin, and PI3K.^29^ The present study found that OPN gene overexpressed in three subtypes of NSCLC and was correlated with high metastasis and mortality rates of patients with NSCLC.

Roles of OPN in the development and repair of multiple cells/tissues as well as OPN‐involved mechanisms vary among organs, species, diseases, severities, and durations.^27^, ^30^ The present study validated the importance and variation of selected OPN between normal bronchial epithelial cells and cancer cells, responses to different pathological challenges, roles of internal and external origins, and correlations with prognosis and metastasis. We found that the expression of OPN gene was downregulated by the “second hit” like LPS, hypoxia, and secretion from activated normal epithelial cells. The two‐hit model is suggested to exist in many pathological conditions, where the “first hit” is the primary condition of the disease and the “second hit” presents additional challenges during the disease, for example, infection, hypoxia, reperfusion, and chemicals, leading to cell/tissue damage, genetic mutation, immune reprograming, and organ dysfunction.^31^, ^32^ Our data demonstrated that OPN gene expression increased after stimulation with CSE or the “second hit,” while cell proliferation reduced correspondingly, of which responses could be prevented by the deletion of OPN gene. OPN gene and protein overexpressed in NSCLC tissue and lung cancer cells treated with smoking. The external OPN could decrease cell proliferation of normal airway epithelial cells and downregulate the mRNA expression of most PIK3 subgroups, while increasing proliferation of lung cancer cells. OPN biological and pathological roles vary among cells, diseases, and severities due to the interaction of OPN with other molecules and heterogeneity of OPN per se during evolution.^27^ OPN could contribute to intra‐ or inter‐tumoral heterogeneity, complexity, progression, metastasis, sensitivity, and response of lung cancer cells to therapy, although molecular mechanisms remain unclear.

OPN played decisive roles in lung cancer cell proliferation, movement, metastasis, and EMT formation. Our data indicate that OPN may contribute to the metastasis and recurrence of lung cancer cells and influence patient prognosis through altered EMT processes. EMT was found to be an important regulator in the transit from chronic lung inflammation, remodeling, and fibrosis into lung cancer.^33^ Activated Type‐II or Type‐III EMT might be responsible for the process of smoking‐associated epithelial injury/repair, small airway fibrosis and obstruction, and carcinogenesis, as more than 90% of human cancer arises in epithelia with potential involvement of EMT, probably through the regulation of Wnt/β‐catenin pathway,^34^ p53 up‐acetylation,^35^ PI3K‐dominated signals,^36^ or OPN‐STAT3 pathway.^37^ The present study provides the direct evidence that external OPN could induce the development of lung cancer cell EMT and increase cell migration, movement, and proliferation. Of EMT transcriptional factors, OPN may activate EMT transcriptional networks through the initial signaling of Snail, like in other situations.^38^ Our data demonstrated that OPN silencing could inhibit the tumor growth and the process of EMT. Other roles of OPN need to be furthermore investigated to unravel regulatory effects of OPN in lung cancer.

The OPN‐PI3K pathway can be one of the critical mechanisms by which OPN induced the development of EMT and the migration of lung cancer cells. OPN acted as a secreted phosphor‐glycoprotein to promote lung cancer cells migration and invasion through different pathways.^39^ Of multipathways, PI3K and Erk1/2 have been reported to play important roles in the development of EMT in human cancer cells.^40^, ^41^ Results of the present study indicated that both external and internal OPN protein could regulate the genes of PI3K isoforms in epithelial cells after challenge. Network characteristics and interactions of genes encoding PI3K isoform proteins differed among signal pathways, responsible for cell proliferation and sensitivity to PI3K‐associated or‐specific inhibitors.^42^, ^43^ The mutation of p53 could lead to secondary changes in chemical and biological natures of PI3K subunit proteins or intercommunication between p53 and PI3K isoform genes. On the other hand, OPN could regulate p53 expression; for example, p53 was upregulated in OPN deletion and downregulated in OPN accumulation in normal airway epithelial cells, while p53 was upregulated in lung cancer cells. We found that OPN‐PI3K or OPN‐MEK pathways could play critical roles in regulation of EMT development and migration/movement of lung cancer cells through the regulation of vimentin‐dependent signals. EMT was associated with proliferation, invasion, and metastasis through transcriptional and epigenetic regulations, gene regulatory network and interaction, and circulating mediators/receptors (e.g., lncRNA, HGF, and met).^44^, ^45^, ^46^ It is also possible that EMT might develop during lung cancer cell movement and proliferation increased by internal and external OPN, as cell movement and proliferation increased earlier than EMT occurrence. In addition to EMT contributions to the development of metastasis, the process of metastasis may accelerate the formation of EMT, although it needs to be further explored and confirmed. Vimentin may be the critical regulator of lung cancer cell movement, metastasis, and EMT through OPN‐PI3K and/or OPN‐MEK pathways. Dynamics and function of vimentin as a decisive element of the cell cytoskeleton in migrating cells are responsible for the process of cell migration and for the invasive behaviors of metastatic cancer cells by altering microtubules and actomyosin networks.^47^ PI3K and MEK inhibitors may block OPN‐increased intermediate filaments of cell type‐specific fiber networks by downregulating gene expression of vimentin, leading to the poor plasticity and EMT formation of lung cancer cells. However, there are still some deficiencies in this study. Although A549 is a commonly used cell line and a well‐established EMT cell model in lung cancer, there are still some differences between lung cancer cell line and tumor tissues in reality. Additionally, the mechanism between OPN and CSE/hypoxic condition is still a big research field. Cell migration, proliferation, and EMT in the NSCLC cells with cotreatment of OPN and CSE/hypoxic condition in vivo, as well as the interaction of OPN and EMT in EGFR‐TKI‐resistant patients remain to be further studied.

In conclusion, the present study selected OPN as a target molecule in lung cancer from global genomic databases, associated with lymph node metastasis, postresection recurrence/metastasis, and prognosis of patients with OPN‐high expression lung cancer. Biological behaviors and pathological responses of OPN varied among diseases, challenges, and severities. OPN mRNA and proteins overexpressed in lung cancer tissue, which correlated with the existence of EMT. Internal and external OPN play decisive roles in lung cancer cell movement, proliferation, and EMT formation, through the upregulation of OPN‐PI3K and OPN‐MEK pathways. PI3K and MEK inhibitors downregulated the process of EMT and biological behaviors of lung cancer cells, probably through alteration of vimentin‐associated cytoskeletons. Thus, our data indicate that OPN can be a metastasis‐associated or ‐specific biomarker for lung cancer and a potential target for antimetastatic treatment.

## FUNDING INFORMATION

Operation Funding of Shanghai Institute of Clinical Bioinformatics and Shanghai Engineering and Technology Center for Artificial Intelligence of Lung and Heart Diseases, Zhongshan Hospital; National Nature Science Foundation of China, Grant Numbers: 81800034, 81873409; Outstanding Youth Foundation of Zhongshan Hospital, Grant Number: 2018ZSQN01; National Key Research and Development Program of Precision Medicine, Grant Number: 2017YFC0909500.

## CONFLICT OF INTEREST

The authors declare that there is no conflict of interest.

## AUTHOR CONTRIBUTIONS

Lin Shi, Jiayun Hou, and Lin Wang designed the study and completed the experimental process and literature search. Yiwen Zhang, Xiangdong Wang, and Yuanlin Song wrote and edited the manuscript. Lin Shi, Huirong Fu, and Xiangdong Wang completed generation of figures. Jiayun Hou, Lin Wang, and Yuanlin Song performed bioinformatics and statistical analysis. All authors reviewed the manuscript and read and approved the final version.

## Supporting information

SUPPORTING INFORMATION.
Supplement Table S1 Sequences mentioned in the article
