# Supplementary material for: Regulatory roles of osteopontin in human lung cancer cell epithelial‐to‐mesenchymal transitions and responses
Source: Clin Transl Med. 2021 Jul 8;11(7):e486. doi: 10.1002/ctm2.486 (PMC8265167; doi:10.1002/ctm2.486)
Supplement: Supplementary file 1 — SUPPORTING INFORMATION. Supplement Table S1 Sequences mentioned in the article [file CTM2-11-e486-s001.pdf]

Supplementary Table 1: Sequences mentioned in the article

Name	Sequences	
	**Forward primer**	**Reverse primer**
OPN	5’- AGTGATTTGCTTTTGCCTCCT-3’	5’- GCTTTCGTTGGACTTACTTGGA-3’
E-cadherin	5’-CCCACCACGTACAAGGGTC-3’	5’-CTGGGGTATTGGGGGCATC-3’
Vimentin	5’-CGCCAGATGCGTGAAATGG-3’	5’-ACCAGAGGGAGTGAATCCAGA-3’
Snail	5’-AATCGGAAGC CTAACTACAGCG-3’	5’-GTCCCAGATGAGCATTGGCA-3’
Twist	5’-GTCCGCAGTCTTACGAGGAG-3’	5’-GCTTGAGGGTC TGAATCTTGCT-3’
GAPDH	5’-GATGCTGGCGCTGAGTACG-3’	5’-GCTAAGCAGTTGGTGGTGC-3’
siRNA- OPN1	5‘--GTTTCACAGCCACAAGGAC--3’	5‘--GTCCTTGTGGCTGTGAAAC--3’
siRNA-OPN2	5‘--GCGAGGAGTTGAATGGTGCATACAA--3’	5‘--TTGTATGCACCATTCAACTCCTCGC--3’
siRNA-OPN3	5‘--CGACTCTGATGATGTAGATGACACT--3’	5‘—AGTGTCATCTACATCATCAGAGTCG--3’
siRNA-NC	5’--CACTGAACTACTCATGGTGTGAGAT --3’	5‘--ATCTCACACCATGAGTAGTCTAGTG --3’
PIK3CA	5’-GGGATGATTTACGGCAAGATA-3’	5’-CCACACAGTCACCGATTGA-3’

PIK3CB	5'-TGCGACAGATGAGTGATGAA-3'	5'-TCCTCCGATTACCAAGTGCT-3'
PIK3CD	5'-GCCAACATCCAACTCAACAA-3'	5'-CCACACAATAGCCAGCACAG-3'
PIK3C2A	5'-GATTACCTGGGCCTTCCAC-3'	5'-AGTGGGCATTCTTGGATTGA-3'
PIK3C2B	5'-TCCACCTTGAACTACCTCGTC-3'	5'-AAGTCTCCATCAGCCAGCAG-3'
PIK3C2G	5'-CTCCTGGCATCAAGTTAGCA-3'	5'-TCTGGAATCATCAGCACCAT-3'
PIK3C3	5'-ATCCCGTTGCCTTTAGAACC-3'	5'-TGCCTCCATCTTCCGTCTTA-3'
PIK3R1	5'-GGACGGCGAAGTAAAGCAT-3'	5'-TGACATTGAGGGAGTCGTTG-3'
PIK3R2	5'-GATGGGCACTATGGCTTCTC-3'	5'-TGCTGGTATTTGGACACAGG-3'
PIK3R3	5'-AGCACAACGACTCCCTCAAC-3'	5'-AAATGCCAGAGAACCACCTC-3'
PIK3R4	5'-TGGCATTTGTGTCCCTTTGT-3'	5'-TGCTGGATGAGTTGCTGAAG-3'
TP53	5'-TGAAGCTCCCAGAATGCCAG-3'	5'-GCTGCCCTGGTAGGTTTTCT-3'

siRNA-OPN-1, -2, -3 are three kinds of OPN-siRNAs targeted on different sequences. siRNA-NC is a negative control siRNA with the same nucleotide composition but which lacks significant sequence homology to OPN.
